# Doxycycline Attenuates Pig Intestinal Microbial Interactions and Changes Microbial Metabolic Pathways

**DOI:** 10.3390/ani13081293

**Published:** 2023-04-10

**Authors:** Jiaojiao Xu, Jiadi Liang, Wenjun Chen, Xin Wen, Na Zhang, Baohua Ma, Yongde Zou, Jiandui Mi, Yan Wang, Xindi Liao, Yinbao Wu

**Affiliations:** 1 Guangdong Laboratory for Lingnan Modern Agriculture, College of Animal Science, South China Agricultural University, Guangzhou 510642, China; 2 National Engineering Research Center for Breeding Swine Industry, South China Agricultural University, Guangzhou 510642, China; 3 Guangdong Provincial Key Laboratory of Agro-Animal Genomics and Molecular Breeding, South China Agricultural University, Guangzhou 510642, China; 4 Foshan Customs Comprehensive Technology Center, Foshan 528200, China; 5 Maoming Branch, Guangdong Laboratory for Lingnan Modern Agriculture, Maoming 525000, China

**Keywords:** doxycycline, pig, intestinal microbial community, 16S rRNA sequencing, KEGG

## Abstract

**Simple Summary:**

The stability and balance of the intestinal bacterial community is the foundation of animal health. It is unclear how the therapeutic dose of doxycycline affects pig intestinal bacterial ecology. In our study, the effect of high doxycycline concentration on the bacterial community was not reflected in the diversity of the microbial community structure but in the bacterial interaction. Moreover, the functional prediction also showed that bacterial interaction may be related to changes in metabolic pathways. This study provides a reference for the effect of therapeutic doxycycline on the pig intestinal bacterial community in pig breeding.

**Abstract:**

Doxycycline is a therapeutic veterinary antibiotic commonly used in pig breeding. In this study, 27 fattening pigs of 33.5 ± 0.72 kg were divided equally into 3 groups. Doxycycline at 0, 3, and 5 mg/kg body weight was added to the feed in groups CK, L and H. The medication and withdrawal periods were set at 5 and 28 days. The results showed that the doxycycline average concentrations in groups L and H during the medication period were 117.63 ± 13.54 and 202.03 ± 24.91 mg/kg dry matter, respectively. Doxycycline levels were lower than the detection limit after 20 days. Doxycycline did not affect the diversity of the intestinal microbial community structure. The relative abundances of *Streptococcus* were significantly higher in treatment groups than that in group CK, and *Alishewanella*, *Vagococcus*, *Cloacibacterium*, and *Campylobacter* abundances were significantly positively correlated with doxycycline concentration. Interestingly, the microbiota cooccurrence network suggested that high doxycycline concentration weakened the interactions among bacteria until day 33. Functional prediction showed that doxycycline significantly altered metabolic pathways related to the cell membrane. The results revealed that the use of doxycycline during pig breeding can affect bacterial abundance during the withdrawal period, and it may affect interactions among bacteria and change the intestinal metabolic pathways.

## 1. Introduction

With the expansion of livestock and the poultry breeding scale, the usage of veterinary antibiotics is increasing. It was reported that the amount of antibiotics used in food animals was expected to be 93,309 tons in 2017, and that it might rise by 11.5% to over 100,000 tons in 2030 [1]. According to the announcement from the Ministry of Agricultural and Rural Affairs of the People’s Republic of China in 2019, as the world’s largest veterinary antibiotic producer and consumer, China used 41,967 tons of antibiotic in 2017, accounting for 45% of global use. The veterinary antibiotics used in livestock and poultry were found to be mainly sulfonamides, fluoroquinolones, tetracyclines, trimethoprim, metronidazole, β-lactamases, and macrolides [2]. Although antibiotics were abandoned as growth-promoting feed additives in China in 2020, they can be used as therapeutic medicines.

Doxycycline is a semisynthetic second-generation tetracycline antibiotic and one of the most commonly used antibiotics worldwide [3,4]. Doxycycline plays a pharmacological role by inhibiting the protein synthesis of bacteria by binding with the 30S subunits of ribosomes and preventing the attachment of aminoacyl-tRNA [5]. Presently, doxycycline is widely used in livestock and poultry production in China, accounting for 69.67% of the total amount of tetracyclines, and its usage in the pig industry and chicken industry reached 2300 tons and 786 tons, respectively [6]. The residue rate of doxycycline in laying hen feces was as high as 95.72% [7], which could cause great environmental pressure.

The use of antibiotics can change the intestinal microbial community structure and diversity. Intestinal microorganisms play an important role in maintaining the health of animal hosts as a natural barrier against pathogens, and can assist the host in extracting nutrients for the development of the immune system and epithelial cells [8]. Boynton [9] found that doxycycline reduced the richness and diversity of microbial communities in mouse feces, resulting in a decrease in the relative abundance of Firmicutes and an increase in the relative abundance of Bacteroidetes. Another study showed that the abundance of Bifidobacterium, a genus of tetracyclines resistant bacteria, increased significantly in the feces of patients during doxycycline treatment [10]. The microbial phenotypes and abundance in pig feces changed after the application of antibiotic compounds for 14 days in 18-week-old piglets without previous antibiotic exposure [11]. Moreover, changes in intestinal microorganisms might affect host metabolism and immune function. When zebrafish were exposed to tetracycline for a long duration, the expression of genes in the liver was significantly upregulated, which led to the dysregulation of some metabolic pathways; this may be related to the intestinal microbial disturbance caused by tetracycline [12].

However, the actual effect of applying doxycycline at the therapeutic dose on the intestinal microorganisms of livestock is still unclear. It is worth tracking the dynamic changes in the microbial community after administering antibiotics for the treatment of livestock. Therefore, this study aimed to explore the degradation of doxycycline and its potential influence on the intestinal microbial structure when the use of doxycycline is regulated.

## 2. Material and Methods

### 2.1. Experimental Design

A total of 27 Duroc × Landrace × Yorkshire crossbred fattening pigs of 33.5 ± 0.72 kg were divided into 3 groups with 9 pigs in each group. Based on the guidelines of the “Veterinary Pharmacopoeia of the People’s Republic of China”, doxycycline at 3 mg/kg body weight (BW) and 5 mg/kg BW was added to the feed for group L and group H, respectively. The blank group (group CK) did not receive any veterinary antibiotics. The medication and withdrawal periods were set at 5 days and 28 days, respectively. Before the formal experiment, an advanced period of 10 days was set to ensure that the physical state of pigs was normal. Individual metabolic cages were used for each pig to ensure the full collection of feces and residual feed. Completed feed was purchased from a feed company (Guangdong, China), and there were no veterinary antibiotics in the feed during the advanced and withdrawal periods. The properties of the complete feed were shown in Table S1 (in the Supplementary Materials). The pig feces were collected at 9:00 and 17:00 each day during the first week of the formal experiment (days 0–7) and at 17:00 each day during the following 26 days. The feces of each pig in each group were collected separately in individual sampling bags. Then, the feces samples were stored in a 4 °C refrigerator for the determination of doxycycline concentrations and microbial community structure.

### 2.2. Determination of Doxycycline Concentration

The concentration of doxycycline in pig feces and the weight of feces were measured every day to assess residual doxycycline levels. The determination of doxycycline levels was based on a previously described method [13] with corresponding adjustments. The specific operation method was listed in Text S1. The calculation formula of the residue rates of doxycycline in pig feces was equal to the ratio of doxycycline residues to the total feeding.

### 2.3. Total DNA Extraction and 16S rRNA Gene Sequencing

The total microbial DNA in feces on days 0, 1, 5, 8, 15, and 33 was extracted using an Omega E.Z.N.A. TM Soil DNA Kit (Omega, Norcross, GA, USA). Six samples from the three groups were randomly selected on the same day (*n* = 108, repeats = 6). To analyze the bacterial community structure and diversity, the universal 16S rRNA gene V3-V4 regions were amplified with the primers (357F:5′-ACTCCTACGGGAGGCAGCAG-3′, 806R:5′-GGACTACHVGGGTWTCTAAT-3′). PCR products were detected by electrophoresis with a 2% agarose gel. Equal amounts of samples were mixed according to the concentration of PCR products. After full mixing, 2% agarose gel electrophoresis was used to detect the PCR products. The product was recovered by a gel recovery kit for the target strip. A TruSeq DNA PCR-Free Sample Preparation Kit (Illumina, CA, USA) was used for library construction. The constructed libraries were quantified by Qubit and q-PCR. The qualified libraries were sequenced by a NovaSeq 6000 (Illumina, CA, USA). After the barcode and primer sequences were removed, FLASH software (V1.2.7) was used to assemble the reads of the samples to obtain raw tag data. QIIME software (V1.9.1) was used to truncate raw tags from the first low-quality base site with the continuous low-quality base value (threshold of 19) reaching 3, and filtering out the tags whose continuous high-quality base length was less than 75% of the tag length to obtain clean tags. Vsearch (V2.15.1) was used to remove chimeric sequences. Denoising was performed to predict biological sequences and filter chimeras to obtain initial amplicon sequence variants (ASVs). Vsearch (V2.15.1) was used to remove chimeras to obtain feature sequences and remove plastid and non-bacterial sequences. Finally, Vsearch (V2.15.1) was used for the standardization of equivalent sampling to obtain classified information.

### 2.4. Data Analysis

The data were analyzed by Excel 2019 and SPSS 26.0. One-way ANOVA was performed to analyze the concentration of doxycycline and physiochemical properties. The α-diversity of the bacterial community, including the Chao1 and Shannon indexes, was calculated using QIIME. PCoA was performed by R studio (V4.0.3) to assess the β-diversity of the bacterial community. The difference in the abundance of bacterial phyla and genera was assessed by LEfSe analysis. Network analysis based on the Spearman coefficients based on the levels of bacterial genera was performed by Gephi 0.9.2. The functional prediction of KEGG pathways was performed by PICRUSt2.

## 3. Results

### 3.1. Trends in the Residual Concentration of Doxycycline

**Figure 1:**
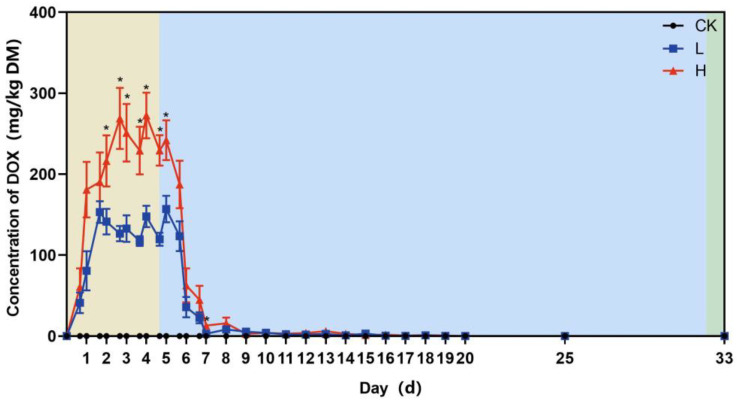
The concentration of doxycycline in pig feces. The bars represent the standard error. The * indicates that the concentration of doxycycline in group H is significantly different from it in group L (*p* < 0.05). The medication period was defined as 0–4 days for 5 days in total (light yellow color), and the withdrawal period was defined as 5–32 days for 28 days in total (blue color). The 33rd day was the first day after the withdrawal period (green color).

The residual concentration of doxycycline in pig feces was shown in Figure 1. During the treatment period, the concentration of doxycycline in the pig feces in both groups L and H increased rapidly and was maintained during the second to fourth days of the treatment period and on the fifth day (the first day of the withdrawal period). The concentration of doxycycline in group L exhibited a peak value of 157.05 ± 16.43 mg/kg dry matter (DM) on the fifth day, and that in group H peaked at 272.49 ± 28.24 mg/kg DM on the fourth day. The average concentrations of doxycycline in group L and group H were 117.63 ± 13.54 and 202.03 ± 24.91 mg/kg DM, respectively, on day 6 (day 2 of the withdrawal period). Subsequently, the concentration of doxycycline in groups L and H decreased rapidly to 35.92 ±12.68 mg/kg DM and 62.72 ± 20.95 mg/kg DM, respectively. Then, doxycycline was present at low concentrations ranging from 0.31 to 8.26 and 0.66 to 15.87 mg/kg DM in the pig feces of groups L and H, respectively, until the limit of detection (LOD) was reached on day 20. The average concentrations of doxycycline during the withdrawal period in groups L and H were 23.45 ± 10.31 and 37.89 ± 16.02 mg/kg DM, respectively. The residue rates of doxycycline in pig feces were 21.62 ± 1.96% and 23.75 ± 2.90% in groups L and H, respectively, during the medication period, and 12.13 ± 1.18% and 14.09 ± 1.03%, respectively, during the withdrawal period (*p* > 0.05). The total residues were 177.16 ± 14.55 mg/per pig and 331.09 ± 23.61 mg/per pig, and the total residue rates were 33.75 ± 2.77% and 37.84 ± 2.70%, respectively (*p* > 0.05). The results indicated that applying different concentrations of doxycycline could not affect the residue rate in pig feces.

### 3.2. Diversity of the Bacterial Community

A total of 9,576,413 reads of raw tags were obtained, and the numbers of clean tags and effective tags were 8,468,458 reads and 6,694,648 reads, respectively. The average effective rate was 70.14%. A total of 5608 ASVs were finally acquired.

**Figure 2:**
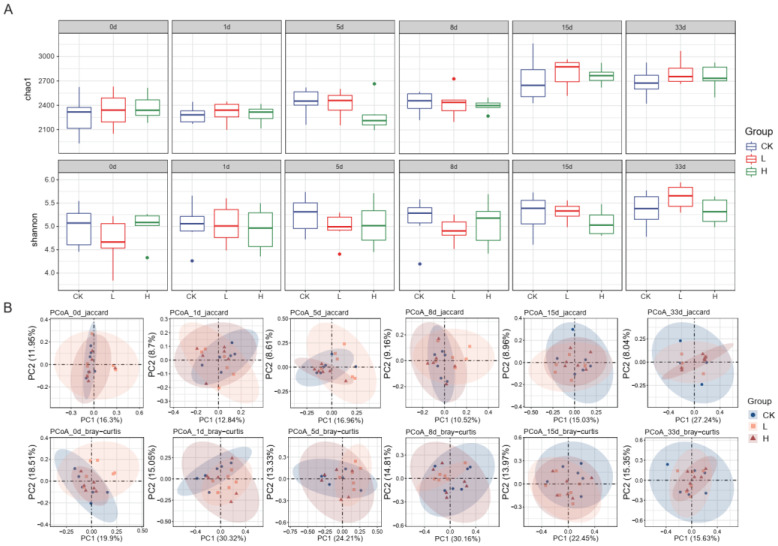
(**A**): The α-diversity of the bacterial community, including Shannon and Chao1 indexes in pig feces; the two indexes of each group on days 15 and 33 were significantly increased compared to on days 0, 1, 5 and 8 in each group (*p* < 0.05). (**B**): The β-diversity of the bacterial community in pig feces by PCoA based on binary Jaccard distance and Bray–Curtis distance; the confidence ellipse was set at the 95% level based on the β-diversity of samples on the same day.

The α-diversity of the bacterial community including the Shannon and Chao1 indexes in pig feces was shown in Figure 2A. Compared with day 0, the two indexes in each group on days 0, 1, 5 and 8 were similar but significantly increased on days 15 and 33 in each group (*p* < 0.05), indicating that the richness and diversity of the bacterial community in each group increased as age increased. However, there was no significant difference in these indexes among all groups on the same day (*p* > 0.05), which indicated that doxycycline feeding had no significant effect on the α-diversity of the bacterial community structure in pig feces.

The β-diversity of the bacterial community determined by PCoA based on binary Jaccard and Bray–Curtis distances in pig feces is shown in Figure 2B. The Jaccard distance was calculated according to species clustering, and the Bray–Curtis distance was calculated based on the presence and relative abundance of species. As shown, there were no significant differences among the treatment groups, and the bacterial community on the same day in all groups was clustered. Significant differences were observed between samples from different days (Figure S1).

### 3.3. Bacterial Community Structure at the Phylum and Genus Levels

**Figure 3:**
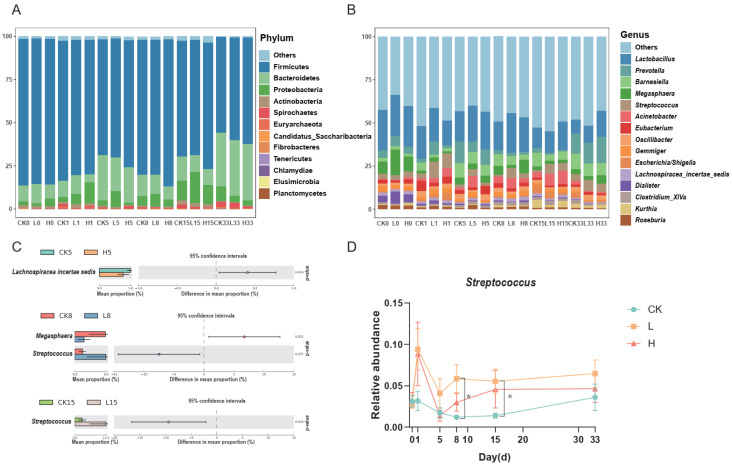
The bacterial community structure at the phylum level (**A**) and genus level (**B**) in pig feces. (**A**) The relative abundance of 12 phyla was 98.22%. (**B**) The top 15 genera accounted for 54.12% of the total relative abundance; there were 11 bacteria belonging to Firmicutes, including *Lactobacillus*, *Megasphaera*, *Streptococcus*, *Eubacterium*, *Oscillibacter*, *Gemmiger*, *Lachnospiracea_incertae_sedis*, *Dialister*, *Clostridium_XlVa*, *Kurthia* and *Roseburia. Prevotella* and *Barnesiella* which belong to Bacteroidetes, and the remaining *Acinetobacter* and *Escherichia/Shigella* belong to Proteobacteria. (**C**) The bacteria in the treatment groups were significantly different from those in the group CK and belonged to the top 15 genera. (**D**) Line chart of the relative abundance of *Streptococcus* over time (* represents *p* < 0.05).

During the whole experiment, Firmicutes was the most dominant phylum in all groups, followed by Bacteroidetes and Proteobacteria. The relative abundance of the three dominant phyla accounted for at least 92.63% (Figure 3A). The relative abundance of Firmicutes showed a decrease during the whole process, and the proportion of its abundance decreased from 84.59% to 58.70% on average on days 0 and 33. However, the relative abundance of Bacteroidetes continued to increase and it was 3.41 times higher on day 33 than on day 0 on average, with the relative abundance ratio increasing from 9.40% to 34.12%. The relative abundance of Proteobacteria on day 15 was at the maximum level, reaching 13.43%. However, Firmicutes was still the most dominant phylum. There were no significant differences in phylum abundances among all groups on the same day, indicating that doxycycline administration did not affect the bacterial community at the phylum level.

The relative abundances of the top 15 bacteria at the genus level were shown in Figure 3B. Most of the genera belonged to Firmicutes, and *Lactobacillus* was found to have the highest abundance during the whole experiment, accounting for 8.11–25.50% of the bacteria. There were three significantly different bacteria at days 5, 8, and 15 (Figure 3C). Moreover, the relative abundance of *Streptococcus* in group L was significantly higher than that in group CK on days 8 and 15 (*p* < 0.05). Then, the *Streptococcus* abundance in different treatment groups was tracked (Figure 3D). The relative abundance of *Streptococcus* increased on day 1 after doxycycline treatment in groups L and H and decreased rapidly after doxycycline treatment was stopped on day 5. Then, the *Streptococcus* abundance in groups L and H continued to increase and was higher than that in group CK from day 5 to day 33.

### 3.4. Correlation between Doxycycline Concentration and Relative Abundance of Microorganisms

**Figure 4:**
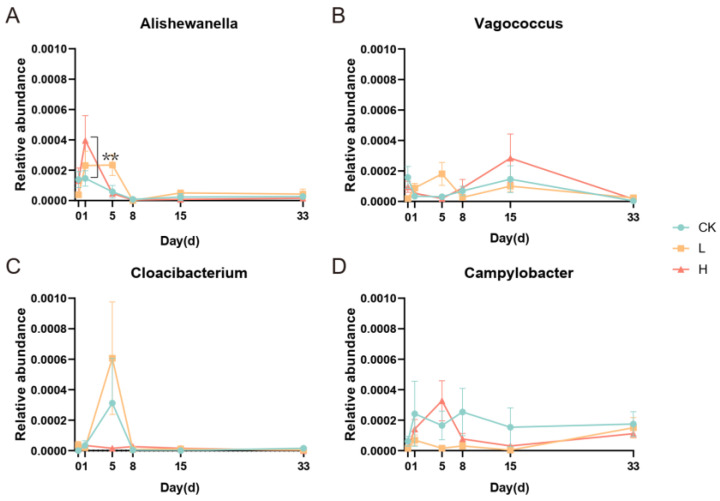
The relative abundance of four bacteria significantly associated with doxycycline concentration (** represents *p* < 0.01). The four bacteria were *Alishewanella* (**A**), *Vagococcus* (**B**), *Cloacibacterium* (**C**), and *Campylobacter* (**D**).

The bacterial genera with a significant correlation with doxycycline concentration in different treatment groups were screened, and four bacteria with a positive significant correlation with doxycycline concentration were identified (Figure 4). An analysis of the correlation between bacterial abundance and doxycycline concentration was performed according to Pearson’s correlation coefficient (|r| > 0.5, *p* < 0.05, Table S4). In group L, *Alishewanella*, *Vagococcus*, and *Cloacibacterium* abundances were positively correlated with the doxycycline concentration (*p* < 0.05). In group H, *Campylobacter* was positively correlated with the doxycycline concentration (*p* < 0.01). It is worth noting that the relative abundances of *Alishewanella*, *Cloacibacterium*, and *Vagococcus* all showed an increasing trend on day 5 (the first day of the drug withdrawal period) in group L compared with group CK, which may be related to the doxycycline concentration in group L. On day 5, the concentration of doxycycline in group L was the highest (157.05 ± 16.43 mg/kg) in the whole period. The relative abundance of *Campylobacter* was lower in group H than in group CK (except on day 5), and its abundance started to recover until day 33. *Campylobacter* growth was inhibited by doxycycline treatment.

### 3.5. Cooccurrence Networks among the Bacterial Genera

**Figure 5:**
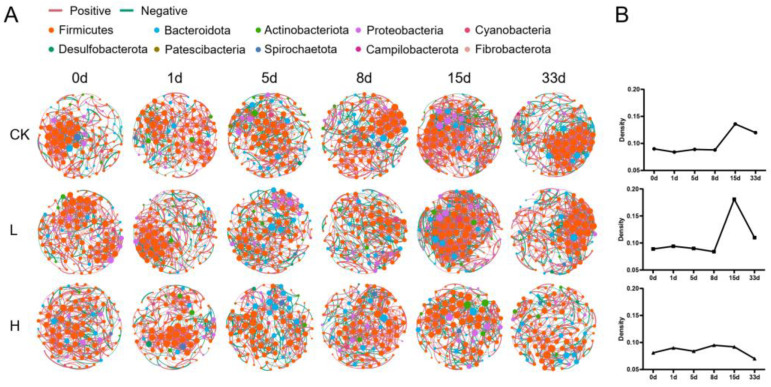
(**A**) Bacterial cooccurrence network depicting Spearman’s correlation coefficient based on the levels of bacterial genera (|r| > 0.8, *p* < 0.05). Nodes represent bacterial genus. The colors indicate the phyla to which they belong, and the sizes indicate the degree of genera, which means the number of connections with other nodes. Edges represent significantly positive (red) or negative (green) correlation, and the widths of edges indicate the Pearson’s correlation coefficient (|r|); (**B**) network density in different groups during the whole experiment.

To probe the potential mechanism underlying the effect of doxycycline on fecal microbiota interactions, the cooccurrence networks among the bacterial genera among all groups were compared. Networks of the top 100 bacterial genera in abundance based on Spearman’s correlation coefficient were shown (Figure 5). The trends of network densities were similar across all groups. Interestingly, the bacterial interactions were weaker in group H but much stronger in group L than in group CK on day 15. The network density in group H decreased to 0.092, while those in groups CK and L increased to 0.136 and 0.181, respectively. Unlike in group CK, the number of negative edges in group H increased from 192 to 243, with the number of positive edges decreasing from 441 to 187. The number of negative and positive edges in group L increased to 333 and 525, respectively. Until day 33, the density in group H (0.07) was still less than those in groups CK (0.12) and L (0.11). Therefore, it seems that doxycycline plays a negative role in the development of bacterial interactions in the pig fecal microbiota.

### 3.6. PICRUSt2 Function Prediction

**Figure 6:**
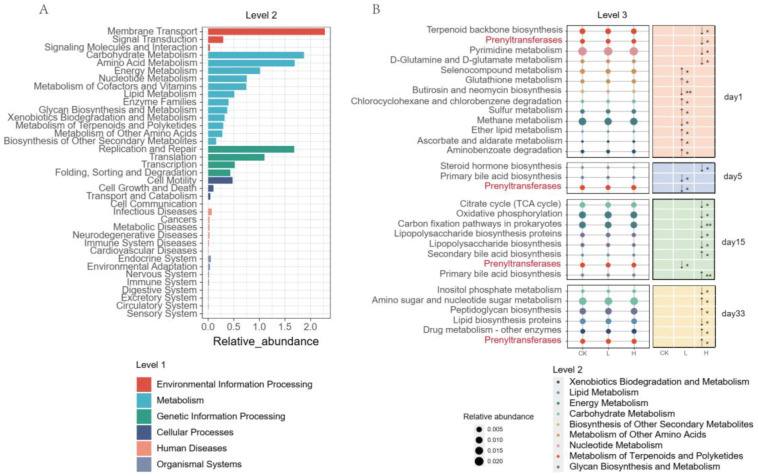
The relative abundance of KEGG level 1, level 2, and level 3 pathways in the three groups according to PICRUSt2 function prediction. (**A**) The bar plot was generated with the relative abundances of all the level 2 pathways, and different colors represent different level 1 metabolic pathways. (**B**) Bubble plots based on the relative abundance of significantly different level 3 metabolic pathways in different groups at different days (*p* < 0.05). The size of the bubble represents the relative abundance of pathways, while the color indicates the level 2 pathway to which it belongs. The prenyltransferases pathway was marked red text colour. The symbol * indicates that the relative abundance of the level 3 pathway in this group was significantly downregulated (↓) or upregulated (↑) when compared with that in group CK (* represents *p* < 0.05, ** represents *p* < 0.01).

To determine the effect of doxycycline feeding on the development of the microbial community in pig manure, PICRUSt2 was used to explore the KEGG pathways in different groups. At level 1, the pathways with higher relative abundances were environmental information processing, metabolism and genetic information processing, and the main pathway was metabolism (Figure 6A). At level 2, these metabolism-related pathways were mainly enriched in carbohydrate metabolism, amino acid metabolism, energy metabolism, nucleotide metabolism, metabolism of cofactors and vitamins, lipid metabolism, enzyme families, glycan biosynthesis and metabolism, xenobiotics biodegradation and metabolism, metabolism of terpenoids and polyketides, metabolism of other amino acids, and biosynthesis of other secondary metabolites.

Further analysis was performed on pathways with significant differences in metabolism-related pathways. One-way ANOVA was conducted for level 3 pathways related to metabolism (Welch’s T test, *p* < 0.05), and the relative abundance of pathways with significant differences in each day was visualized in a bubble plot and is depicted in Figure 6B. There were 26 pathways related to level 3, and they belonged to 9 metabolism-related level 2 pathways, and most of the pathways were found in group H. It is worth noting that the abundance of the prenyltransferases pathway in the doxycycline treatment groups was significantly different from that in the group CK on days 1, 5, 15, and 33. Its relative abundance on day 1 in group H, day 5 in group L, and day 15 in group L was significantly lower than that in the group CK, and its relative abundance on day 33 in group H was significantly higher than that in the CK group (*p* < 0.05). Moreover, on day 1, butirosin and neomycin biosynthesis were downregulated in groups L (*p* < 0.05) and H. On day 5, the primary bile acid biosynthesis pathway in group L was significantly upregulated (*p* < 0.05). On day 15, the primary bile acid biosynthesis and secondary bile acid biosynthesis pathways in group H were significantly upregulated (*p* < 0.05). Bile acids are among the most abundant metabolites synthesized by the intestinal microbiota [14]. Additionally, on day 15, the lipopolysaccharide biosynthesis and lipopolysaccharide biosynthesis proteins pathways were significantly downregulated in group H (*p* < 0.05). Lipopolysaccharide is one of the most studied surface molecules of bacteria and is produced by most gram-negative bacteria [15]. On day 33, the drug metabolism by other enzyme pathways in group H was significantly upregulated (*p* < 0.05), which in group L also showed a trend of upregulation. It is possible that the presence of doxycycline led to a significant upregulation of drug metabolism in the bacterial community.

## 4. Discussion

Doxycycline is a commonly used veterinary antibiotic for the prevention and treatment of diseases in livestock and poultry. In our study, a total of three groups were set up: groups L and H were fed doxycycline at 3 mg/kg BW and 5 mg/kg BW, respectively, and the blank control group was not administered antibiotics. Doxycycline levels fell below the detection limit on day 20 of the experimental period (day 15 of the withdrawal period), which suggested that the drug remained in the animals’ bodies at low concentrations for a long time after oral administration. The reality is that a few antibiotics used in vivo can be fully absorbed and metabolized [16]. Nearly 30–90% of antibiotics, including their metabolites, are released into the environment through feces and urine [17,18]. Although feeding different doxycycline concentrations had no effect on the residue rates in pig feces (Figure 1), it is worth noting that doxycycline has toxicological effects on environmental ecology to a certain extent, along with fecal discharge [19], and it may be enriched in crops through planting substrates and may enter humans and animals through the food chain, eventually threatening human and animal health [20]. Residual antibiotics promote the emergence of antibiotic resistance genes (ARGs) in the environment under antibiotic pressure [21,22], increasing the risk of antibiotic resistance in humans [23,24].

Antibiotics are considered a powerful factor causing an imbalance in animal and human intestinal microbiomes [25,26,27]. Some studies have found that antibiotics can reduce the diversity of animal intestinal flora [28,29]. Another study consistently showed that the core gut microbiome did not change easily [30]. In our study, doxycycline did not change the β diversity of the microbial community structure, and it was consistently random (Figure 2). The reason for the diversity may be the high degree of similarity of the samples, so the effect of the antibiotic on the diversity of the bacterial community was not obvious.

Interestingly, it was found that high doxycycline concentrations had a negative effect on the cooccurrence between bacterial communities, which required a long recovery time (Figure 5). The microbial community structure affected by low doxycycline concentrations could basically be recovered by the end of the withdrawal period. Gao [31] also found that the maturation of the intestinal microbiota was retarded and delayed, and the interaction among bacterial genera was weakened by antibiotic feeding. Compared with the diversity of the bacterial community, the change in the bacterial community was reflected in the composition and interaction inside the flora, not the overall OTU change. In our study, Firmicutes, Bacteroidetes, and Proteobacteria were the most abundant phyla, which was similar to the results in other studies [32,33]. However, long-term residual doxycycline administration changed the bacterial community structure at the genus level. *Streptococcus* belong to Firmicutes, and doxycycline resulted in a continuous increase in the relative abundance of *Streptococcus* with a higher abundance after the drug withdrawal period, and even its abundance in group L was significantly higher than that in group CK during drug withdrawal (*p* < 0.05). *Streptococcus* is an important zoonotic pathogen that causes swine streptococcosis [34]. *Streptococcus* requires a combination of drugs to better kill it because *Streptococcus* can form a strong biofilm and is difficult to remove [35]. In the study, *Streptococcus* has more potential to resist doxycycline and become the dominant bacteria under doxycycline stress compared to other bacteria. This finding indicated that using doxycycline carries the risk of increasing *Streptococcus* abundance in pig feces.

The abundances of the four bacteria were found to be positively correlated with changes in doxycycline concentration (Figure 4). A previous study showed that *Alishewanella* was a denitrifying bacterium [36], and some denitrifying bacteria were important ARG hosts in pig farms based on metagenomic analysis [37]. The increase in *Alishewanella* during the medication period may be due to its resistance to doxycycline, and doxycycline is likely to be used by *Alishewanella* as an energy source (especially carbon and nitrogen sources) [38]. The relative abundance of *Cloacibacterium* in group L was higher on day 5 than that in group CK and then recovered quickly. *Cloacibacterium* is closely related to humans and had been found in many environments [39,40,41]. One clinical study from 2014 to 2019 on *Vagococcus* showed that the isolation of *Vagococcus* was increasing and that *Vagococcus* isolated from patients was resistant to ampicillin, sulfamethoxazole, vancomycin, and many other kinds of antibiotics [42]. High doxycycline concentration played a greater inhibitory effect on *Vagococcus* and *Cloacibacterium*, so these bacteria increased in group L during the medication period. The results showed that the presence of doxycycline inhibited the growth of *Campylobacter* abundance. *Campylobacter* is a major foodborne pathogen; under antimicrobial selection pressure, *Campylobacter* has become increasingly resistant to clinically important antimicrobials [43,44]. Our study showed that doxycycline reduced the risk of resistance transmission in *Campylobacter*.

An early study claimed that antibiotic treatment disrupted intestinal homeostasis and changed the intestinal metabolic pathways critical for host physiology, including bile acid, eicosanoid, and steroid hormone synthesis [45]. In our study, doxycycline was found to significantly change the abundance of some level 3 metabolic pathways, and some of these pathways play important roles in pig fecal microorganisms. The most notable pathway was that involving prenyltransferases, and the other metabolic pathways in the doxycycline treatment groups were significantly different from those in group CK at four time points (*p* < 0.05). At days 1, 5, and 15, the relative abundances of prenyltransferases in the treatment groups were significantly lower than those in group CK. Recent research has shown that prenyltransferases are essential enzymes in bacteria in metabolic pathways and cellular processes, and that they participate in cell wall biosynthesis [46]. Prenyltransferases can catalyze the production of isoprenoids, and isoprenoids play a role in cell wall synthesis, and plasma membrane generation in bacteria, fungi, and animals, including humans [47]. They can be used as potential targets in new drug discovery. Some common antibiotics, namely vancomycin, β-lactams, and bacitracin, play a role in inhibiting the biosynthesis of bacterial cell walls. Doxycycline is a semisynthetic derivative of oxytetracycline that was discovered in 1967. The major targets of doxycycline are ribosome and protein synthesis within the cell, and it prevents the binding of aminoacyl t-RNA to the 30S ribosomal subunit [48]. In our study, prenyltransferases were significantly downregulated in the treatment groups after doxycycline treatment. The results indicated that doxycycline may also inhibit prenyltransferases activity, thus inhibiting bacterial activity. However, this is only an inferred result based on functional prediction, and it needs to be further validated in experiments. On day 15, the lipopolysaccharides pathway was downregulated in group H (*p* < 0.05). Lipopolysaccharides play an important role in forming the cell membrane and the osmotic barrier of gram-negative bacteria, and can protect cells from toxic molecules such as antibiotics and bile salts [15]. Overall, the change in metabolic pathways may inhibit the growth of bacteria and weaken the resistance of the cell membrane, thereby weakening the interaction between bacteria. In addition, the primary bile acid biosynthesis and secondary bile acid biosynthesis pathways were upregulated in the treatment groups. Secondary bile acids can restore intestinal ecology by reinducing inflammation [49], and intestinal microorganisms can use bile acids to control antitumor immunity in the liver [50]. In conclusion, functional prediction suggested that a high doxycycline concentration was more likely to cause changes in the metabolism of the bacterial community and drive the decrease in bacterial interactions.

## 5. Conclusions

Oral administration of doxycycline at different concentrations did not significantly affect the final residue rate of doxycycline in pig feces. Doxycycline did not affect the diversity of the pig intestinal microbial community structure, but the differences in microbial community structure between groups during the withdrawal period explained the delayed effect of doxycycline treatment. The relative abundance of *Streptococcus* increased until day 33, and the *Alishewanella*, *Vagococcus*, *Cloacibacterium*, and *Campylobacter* abundances were significantly positively correlated with doxycycline concentration, and all the bacteria related to drug resistance. Moreover, high doxycycline concentration weakened the bacterial genus interactions until day 33, and doxycycline significantly altered the metabolic pathways related to prenyltransferases, primary bile acid biosynthesis, and lipopolysaccharide biosynthesis sensing-related metabolic pathways. In summary, doxycycline attenuates pig intestinal microbial interactions and changes microbial metabolic pathways.

## Data Availability

The raw data for the 16S rRNA sequencing in this paper are available at the National Center for Biotechnology Information (NCBI). The raw data public accession number is PRJNA848422.

## Supplementary Materials

The following supporting information can be downloaded at https://www.mdpi.com/article/10.3390/ani13081293/s1, Text S1: The detection method of doxycycline; Table S1: The properties of complete feed; Table S2: The mobile phase procedure; Table S3: The mass spectrometry conditions of doxycycline; Table S4: The average daily feces production in three groups; Table S5: The residue rates of doxycycline in three groups during different stages; Table S6: The relative abundance of the bacteria at phylum level; Table S7: The relative abundance of the top 15 bacteria at genus level; Table S8: Genus level bacteria significantly associated with doxycycline concentration. Correlation analysis between bacteria and doxycycline; Figure S1: The β-diversity of bacterial community in pig feces by PCoA based on binary jaccard distance and bray curtis distance.
