# Supplementary material for: Doxycycline Attenuates Pig Intestinal Microbial Interactions and Changes Microbial Metabolic Pathways
Source: Animals (Basel). 2023 Apr 10;13(8):1293. doi: 10.3390/ani13081293 (PMC10135356; doi:10.3390/ani13081293)
Supplement: Supplementary file 1 [file animals-13-01293-s001.zip › animals-2246626-supplementary.pdf]

Doxycycline attenuates pig intestinal microbial interactions and changes microbial metabolic pathways

Supplementary material

Text S1 The detection method of doxycycline

Fecal samples (0.5 g) were added to 5 mL of 0.1 mol/L Na_2EDTA Mcllvaine-methanol extracting solution. Then the mixtures were vortexed for 1 min, and treated with ultrasound for 15 min. After centrifugation (13,000 r/min for 6 min at 4 °C), the supernatants were transferred into a clean tube. The sediments were supplemented with 5 mL of extracting solution again, and the above steps were repeated. The two supernatants were mixed and centrifuged at 13,000 r/min for 6 min at 4 °C. The final supernatants were loaded onto HLB columns that were activated with 3.0 mL of methanol and sequentially equilibrated using 3.0 mL of ddH_2O in advance. Subsequently, the columns were filled with 3 mL of ddH_2O and vacuumized for 1 min. Then, 3 mL of 2% formic acid methanol solution was used for elution, and the eluates were filtered through a 0.22 μm filter membrane and kept at -40°C. Before detection by liquid chromatography–mass spectrometry (LC–MS) (Agilent 1200, Agilent, USA), a small quantity of eluate was diluted to a suitable concentration. A C8 column (4.6 mm × 150 mm, 5 μm, Agilent, USA) was used to separate doxycycline. The temperatures of the column and sample were 30 °C and 20 °C, respectively. The injection volume was 10 μL, and the flow rate was 0.6 mL/min. The mobile phase consisted of a binary mixture of solvents (A: 0.1% aqueous formic acid and B: 0.1% formic acid acetonitrile), and the mobile phase procedure was shown in Table S2. Tandem mass spectrometry was performed in positive ionization mode (ESI+). The capillary voltage was 4500 V. The gas temperature was 300 °C, and the flow rate was 12 L/min. The atomizer voltage was 45 psi. The mass spectrometric conditions were shown in Table S3. The recoveries were 77.87~108.60% and the limit of detection was 10 ppb. The retention time was 7.220 min.

Table S1. The properties of complete feed

Properties	Concentrations
Mosture	9.48 %
Energy	17366.23 kJ/g
Crude protein	199.37 g/kg
Total Nitrogen	31.90 g/kg
Total Carbon	419.50 g/mg
C/N	13.20
Cu	8.33 mg/kg
Zn	50.13 mg/kg

Table S2. The mobile phase procedure

Time (min)	0.1% Acetonitrile of formic acid (%)	0.1% Formic acid (%)
0.0	5	95
3.0	35	65
4.0	85	15
7.0	5	95
9.0	5	95

Table S3 The mass spectrometry conditions of doxycycline

Maternal lon	Lonic ion	Cone voltage	Impact Voltage
445.3	428.1	16	105
	267	16	105

Table S4 The average daily feces production in three groups

**Groups**	**CK**	**L**	**H**
1d	0.7688	0.9788	0.9411
2d	0.7550	0.9088	1.0522
3d	0.8238	0.9450	0.8189
4d	0.9075	0.9325	0.9013
5d	0.7263	1.1275	1.3311
6d	1.0663	0.9988	1.0233
7d	0.8163	1.2100	1.2856
8d	1.3463	1.2163	1.6611
9d	0.7550	0.9925	1.0744
10d	1.0425	1.1563	1.1778
11d	1.1914	1.1113	1.5889
12d	0.9050	1.0138	1.1478
13d	0.8188	1.3463	1.0889
14d	0.7338	0.7775	1.1022
15d	0.9288	0.9313	0.9144
16d	0.8938	1.0975	1.3089
17d	1.0075	1.2800	1.0678
18d	0.6500	1.1343	0.9411
19d	0.7188	0.9313	0.8778
20d	0.8138	0.9250	0.7478
21d	0.6875	0.7375	0.6078
22d	0.9500	1.4538	1.0656
23d	0.9850	1.0725	1.1678
24d	0.7250	0.8513	0.9233
25d	0.9388	1.0788	1.0467
26d	0.9600	1.1538	1.3367
27d	0.7850	1.1650	1.1378
28d	0.9138	1.4175	1.2667
29d	0.9113	1.3863	1.2163
30d	0.9338	1.3788	1.2013
31d	0.9338	1.3788	1.2013
32d	1.0700	1.0638	1.1838
33d	1.2988	1.4450	1.6750

Table S5 The residue rates of doxycycline in three groups during different stages

Groups	CK	L	H
The total feeding doses (mg/per pig)	0	525	875
The residue doses during the medication period (mg/per pig)	0	113.49±10.30	207.79±25.42
The residue doses of doxycycline during the withdrawal period (mg/per pig)	0	63.67±6.22	123.29±8.97
The total residue doses of doxycycline (mg/per pig)	0	177.16±14.55	331.09±23.61
The residue rates during the medication period (%)	0	21.62±1.96	23.75±2.90
The residue rates during the withdrawal period (%)	0	12.13±1.18	14.09±1.03
The total residue rates (%)	0	33.75±2.77	37.84±2.70

Table S6 The relative abundance of the bacteria at phylum level

Phylum	CK0	L0	H0	CK1	L1	H1	CK5	L5	H5	CK8	L8	H8	CK15	L15	H15	CK33	L33	H33
Others	1.4521	1.2627	1.4278	2.5882	2.0553	2.1886	1.6240	1.2922	2.1617	2.1271	2.1315	1.6655	2.5024	1.9759	3.6724	0.6229	0.6940	0.6203
Firmicutes	84.9500	84.5167	84.3167	81.1167	78.3667	77.6833	67.2667	69.0167	73.7667	78.1500	78.0833	85.4167	67.0167	66.7667	73.3833	55.1833	59.3333	61.6000
Bacteroidetes	9.1067	10.8600	8.2333	9.4300	10.8400	4.8333	26.3400	19.2667	20.5500	12.2133	11.0950	7.3367	14.1967	9.9217	9.2667	31.1333	32.4167	32.8833
Proteobacteria	2.1512	1.6373	4.1717	2.6710	6.0467	11.8983	2.7697	9.2995	1.3372	5.6205	7.1983	4.1867	11.4050	18.0483	10.8350	8.5467	3.6000	2.9167
Actinobacteria	1.8785	1.3933	1.5300	1.2637	2.0752	1.7438	1.0977	0.7872	0.4548	0.8422	0.6332	0.6645	2.2950	2.2717	1.9155	0.3163	0.4817	0.5968
Spirochaetes	0.2198	0.1241	0.1050	1.6118	0.1457	0.8336	0.3448	0.1180	1.4103	0.2000	0.3786	0.1612	2.1057	0.6146	0.2229	3.0955	3.0070	1.0902
Euryarchaeota	0.1656	0.1196	0.1062	1.2280	0.3737	0.7084	0.4013	0.1565	0.2194	0.7518	0.2503	0.4613	0.1777	0.2352	0.3483	0.3368	0.3246	0.1692
Candidatus_Saccharibacteria	0.0462	0.0404	0.0327	0.0631	0.0745	0.0815	0.0928	0.0623	0.0577	0.0523	0.1392	0.0423	0.2290	0.1342	0.3327	0.0461	0.0223	0.0177
Fibrobacteres	0.0050	0.0262	0.0115	0.0173	0.0119	0.0065	0.0480	0.0081	0.0188	0.0308	0.0250	0.0123	0.0443	0.0177	0.0108	0.2822	0.0880	0.0980
Tenericutes	0.0031	0.0373	0.0420	0.0046	0.0054	0.0065	0.0038	0.0058	0.0111	0.0035	0.0058	0.0019	0.0042	0.0050	0.0054	0.4058	0.0158	0.0092
Chlamydiae	0.0054	0.0038	0.0054	0.0031	0.0062	0.0035	0.0065	0.0173	0.0058	0.0104	0.0496	0.0284	0.0023	0.0050	0.0023	0.0035	0.0004	0.0008

Elusimicrobia	0.0100	0.0004	0.0008	0.0019	0.0000	0.0000	0.0008	0.0012	0.0027	0.0058	0.0050	0.0027	0.0008	0.0015	0.0004	0.0250	0.0139	0.0027
Planctomycetes	0.0008	0.0008	0.0019	0.0019	0.0004	0.0019	0.0054	0.0054	0.0031	0.0092	0.0065	0.0104	0.0035	0.0023	0.0004	0.0123	0.0050	0.0008

Table S7 The relative abundance of the top 15 bacteria at genus level

Genus	CK0	L0	H0	CK1	L1	H1	CK5	L5	H5	CK8	L8	H8	CK15	L15	H15	CK33	L33	H33
Lactobacillus	23.50	23.88	23.50	18.84	19.41	14.57	17.66	20.63	21.88	16.84	22.95	20.10	11.86	12.58	16.49	8.11	9.67	15.30
Prevotella	4.37	5.99	4.25	2.14	7.65	1.57	12.39	6.10	8.74	2.18	1.92	1.40	2.86	1.02	0.88	11.53	12.25	15.08
Barnesiella	2.07	1.82	1.50	4.77	1.54	1.80	8.36	6.62	6.49	6.85	5.21	3.13	7.72	3.48	4.75	9.04	7.40	6.99
Megasphaera	7.09	14.85	8.61	1.45	2.49	1.13	4.18	3.37	4.09	5.03	1.72	7.68	2.31	2.04	1.81	3.05	1.96	5.10
Streptococcus	3.09	2.73	3.34	3.17	9.40	8.85	1.73	4.08	1.37	1.21	5.87	2.99	1.38	5.54	4.57	3.60	6.46	4.64
Acinetobacter	0.55	0.39	1.44	1.38	2.65	4.64	1.45	6.29	0.54	3.58	5.96	2.39	3.05	6.62	7.81	2.40	0.76	0.93
Eubacterium	1.90	1.23	2.30	5.71	2.24	4.22	2.17	1.57	4.63	2.75	1.76	2.32	1.17	1.08	1.50	1.17	1.42	0.97
Oscillibacter	1.62	1.36	1.58	3.44	1.47	2.28	2.73	1.29	3.31	2.36	1.54	2.18	1.86	1.33	1.08	2.61	3.09	2.49
Gemmiger	3.16	2.19	2.20	2.05	3.07	3.07	1.62	2.80	1.41	2.15	2.32	2.95	1.32	1.38	2.26	0.56	0.56	0.44
Escherichia/Shigella	0.43	0.31	0.58	0.48	1.80	4.29	0.32	0.65	0.24	1.07	0.33	0.66	4.60	3.84	1.21	3.50	0.70	1.25
Lachnospiracea_incertae_sedis	1.80	1.45	1.85	1.56	1.85	1.66	1.01	1.28	0.76	1.81	1.69	2.27	1.81	1.25	1.63	0.64	0.74	0.46
Dialister	4.04	6.80	4.58	0.87	1.72	0.74	0.70	0.70	0.85	0.82	0.32	1.10	0.45	0.28	0.44	0.19	0.19	0.06
Clostridium_XlVa	0.86	0.83	0.70	0.78	1.03	0.72	1.43	1.64	1.19	1.87	1.94	1.80	1.46	2.50	1.33	2.15	1.06	0.84
Kurthia	0.75	0.42	1.17	0.34	0.53	0.72	0.18	0.36	0.25	1.31	0.74	0.67	4.82	1.17	4.03	2.69	1.40	2.14
Roseburia	2.29	1.95	2.13	1.04	1.67	1.21	0.75	2.42	0.61	1.00	1.31	1.45	0.64	1.05	0.91	0.59	0.61	0.40
others	42.46	33.82	40.29	51.96	41.49	48.59	43.33	40.18	43.62	49.22	44.45	46.92	52.68	54.82	49.30	48.19	51.72	42.89

Table S8. Genus level bacteria significantly associated with doxycycline concentration. Correlation analysis between bacteria and doxycycline (according to the Pearson's correlation coefficient (|r|>0.5, $P<0.05$)).

Source	Target	Correlation	P-value	Group
Doxycycline	*Alishewanella*	0.6644	0.0002	L
Doxycycline	*Vagococcus*	0.5361	0.0108	L
Doxycycline	*Cloacibacterium*	0.5305	0.0123	L
Doxycycline	*Campylobacter*	0.5714	0.0055	H

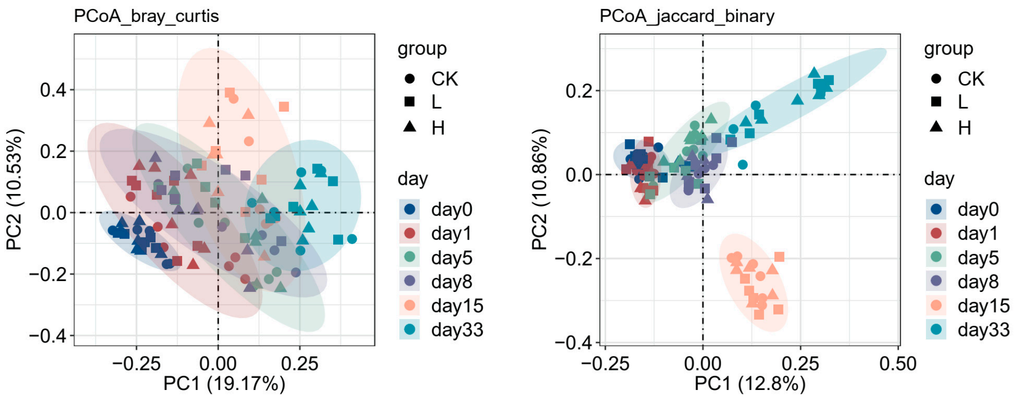

Figure S1. The β-diversity of bacterial community in pig feces by PCoA based on binary jaccard distance and bray curtis distance; Confidence ellipse was set at 95% level based on the β-diversity of samples on the same day.
